# A Pilot Study on the Prevalence of Micronutrient Imbalances in a Dutch General Population Cohort and the Effects of a Digital Lifestyle Program

**DOI:** 10.3390/nu14071426

**Published:** 2022-03-29

**Authors:** José Castela Forte, Rahul Gannamani, Pytrik Folkertsma, Saro Kanthappu, Sipko van Dam, Bruce H. R. Wolffenbuttel

**Affiliations:** 1 Department of Clinical Pharmacy and Pharmacology, University Medical Center Groningen, University of Groningen, Hanzeplein 1, 9713 GZ Groningen, The Netherlands; 2 Ancora Health B.V., Herestraat 106, 9715 LM Groningen, The Netherlands; rahul@ancora.health (R.G.); pytrik@ancora.health (P.F.); sipko@ancora.health (S.v.D.); 3 Department of Neurology, University Medical Center Groningen, University of Groningen, Hanzeplein 1, 9713 GZ Groningen, The Netherlands; 4 Department of Endocrinology, University Medical Center Groningen, University of Groningen, Hanzeplein 1, 9713 GZ Groningen, The Netherlands; sarokanthappu@ancora.health (S.K.); bwo@umcg.nl (B.H.R.W.)

**Keywords:** vitamins, micronutrients, deficiencies, screening, general population, lifestyle, prevention

## Abstract

Maintaining an adequate micronutrient status can be achieved by following a complete, diverse diet. Yet, food trends in Western countries show suboptimal consumption of healthy nutrients. In this study, we explored the prevalence of vitamin and mineral imbalances in a general population cohort of Dutch adults and evaluated the effect of a digital lifestyle program on the nutritional status and nutrition health behaviors of these individuals. A micronutrient panel was measured in 348 participants, alongside a dietary assessment. One hundred users subsequently underwent a remeasurement. We identified at least one nutritional imbalance in 301 individuals (86.5%). A total of 80% improved and normalized B6, 67% improved folate, 70% improved B12, and 86% improved vitamin D. Iron abnormalities were corrected in 75% of the participants. In conclusion, this study found that micronutrient deficiencies of easily obtainable vitamins through diet or supplementation such as B vitamins and vitamin D were more prevalent than expected in a Dutch population. This can partly be explained by insufficient consumption of food groups rich in B vitamins. Our preliminary results in those remeasured after a digitally enabled lifestyle intervention show these imbalances can be corrected with adequate behavioral support complemented with supplementation where needed.

## 1. Introduction

Maintaining an adequate micronutrient intake and status can be achieved by following a complete, diverse diet. A balanced diet rich in fruits, vegetables, whole grains, legumes, and nuts, possibly but not necessarily combined with moderate intake of whole foods and animal-source foods, and limited in processed foods in general is protective against many long-term health conditions [1,2,3]. However, large global surveys show consumption of food groups such as nuts and seeds, milk, and whole grains, amongst the richest in essential micronutrients, can be as low as 12% of the optimal levels [2]. In addition, other studies have found the required intake levels of fruit and vegetables to also often not be met in Western populations [4]. Conversely, the consumption of sugar-sweetened beverages, processed meat, and foods rich in unhealthy fats greatly exceeds the optimal daily amounts [2].

This pattern of suboptimal consumption of healthy nutrients and excessive daily intake of unhealthy nutrients has led to an increase in the prevalence of overweight, obesity, and diet-related non-communicable diseases, while micronutrient deficiencies (MNDs) remain prevalent [1,2,5]. Diseases of the past linked to MNDs such as rickets and scurvy have become uncommon, at least in well-fed Western nations [4]. However, evolving research suggests that suboptimal intake of essential nutrients is still prevalent and can influence the risk of non–communicable chronic diseases (NCCDs) [4]. For example, suboptimal intake of folate, B6, and B12 is a risk factor for cardiovascular disease neural tube defects, and colon and breast cancers [4,6,7]. In turn, insufficient levels of vitamin D increase the risk of osteopenia factures (Fletcher, 2002) and have been associated with tumors, cardiovascular disease, and diabetes as well as the risk for neuropsychiatric disorders, autoimmune diseases, and COPD [8,9].

In many cases, predicting in who and which MNDs will develop remains a challenge. The onset of nutritional deficiencies usually occurs slowly over time as a result of many different and interrelated factors such as pathological changes or genetics or the use of medications which can influence the bioavailability of some vitamins [10]. Individuals may have genetic variants which can increase their need for specific nutrients, such as the well-known association between folate and the MTHFR mutation, which is not only relevant in preventing neural tube defects, but also in cardiovascular disease risk [11]. Recent publications have cited genetic variants in relation to several different nutrients such as B12 and vitamin D [12,13]. Moreover, certain behaviors such as alcohol consumption can increase the need for and/or influence the absorption of vitamins including folate, B6, and B12 [14].

Attempts to answer this ongoing problem have consisted, at least in some countries, of educational campaigns, fortification of foods, and an increase in the use of single or a combination of supplements [15]. However, this is a double-edged sword, as it may not reach the actual at-risk target group and, at the same time, may lead other individuals to unknowingly take excessive doses of nutrients, contributing to nutrient toxicity syndromes. For instance, high blood folate levels achieved through supplementation can mask (sub)clinical B12 deficiency, especially in the elderly population [16,17]. Additionally, the impact of such population-level initiatives remains limited by behavioral constraints such as low self-efficacy, motivation, and perceived benefits, as well as limitations from social constructs such as work routine, family habits, and socioeconomic factors [18,19,20]. In recent years, nutrition-related apps have become more widespread, providing users with information, behavioral guidance, and tracking of nutritional behaviors [21,22]. While digital solutions promoting nutritional behavior change and helping individuals make simple health behavior changes could support the prevention of vitamin and mineral imbalances, the literature on the effectiveness of similar interventions remains scarce [23].

Therefore, in the present study, we aimed to, first, explore the prevalence of vitamin and mineral imbalances in a cohort of presumably “healthy”, Dutch general population adults, and, second, to determine the effect of a digital lifestyle program on the nutritional status and nutrition health behaviors of these individuals.

## 2. Materials and Methods

### 2.1. Study Design

Individuals from a general population in the Netherlands who had participated in a digital lifestyle program at Ancora Health, either by their own initiative or through a workplace health and wellness program, were considered for enrollment in this study. As of October 2021, 348 participants had participated in a lifestyle program and had a vitamin and mineral panel measured, as well as a dietary assessment. This panel included vitamin B6, vitamin B12, folate, vitamin D, iron, and markers related to iron status such as ferritin, transferrin, transferrin saturation, and hemoglobin. Of those, 100 users also came for a re-measurement after concluding the program. The study was declared exempt from institutional review board approval through a waiver issued by the Medical Ethical Review Board of the University Medical Center Groningen (UMCG, waiver number: METC#2021/488), given the data were retrospectively retrieved and analyzed.

### 2.2. Measurements of Vitamins, Minerals, and Other Blood Biomarkers

After an overnight fast, blood samples for measurements of several blood biomarkers including the abovementioned vitamins and minerals were collected. Blood samples were collected into tubes and left for clotting before centrifugation. Serum levels of vitamin B6 were measured by high-performance liquid chromatography (HPLC), folate, vitamin B12, and ferritin by an electro-chemiluminescence (ECLIA) assay, vitamin D by a chemiluminescence immune assay (CLIA), transferrin by turbidimetry (from which transferrin saturation was calculated), and hemoglobin, iron, and hemoglobin by photometry. The cut-offs used for each vitamin and mineral are shown in Table 1. Iron deficiency anemia was defined as low hemoglobin (Hb) with a normal mean corpuscular volume and low iron. Overt iron deficiency was diagnosed when iron was low, independent of Hb. Iron overload was considered when iron, ferritin, and transferrin saturation were high [24].

### 2.3. Food Group and Supplement Consumption

Food group consumption was assessed by means of web-based food frequency questionnaires reflecting participants’ weekly food group consumption, filled in upon enrollment in the Ancora program and at remeasurement. Information on supplement consumption for the micronutrients analyzed in this study was also collected as part of the nutrition questionnaire. Thresholds for unusually low or high portion sizes were defined a priori per food group based on the Dutch National Nutritional Guidelines and complemented by expert knowledge or other international guidelines where necessary (Appendix A) [25]. Participants entered the number of portions consumed per week for each food group as multiple choice, to prevent incorrect entries. Supplement information for each of the measured vitamins and minerals in the blood was also collected, with participants also having the possibility to add any other supplements.

### 2.4. Digital Lifestyle Program

The Ancora Health Personal Health Passport (PHP) is a certified Class I medical device which gives individuals insight into their current health status and possible future health risks based on a broad assessment of body, mind, and lifestyle. Participants undergo a physical intake, where blood biomarkers including cardiometabolic markers and genetic susceptibility scores, as well as physical measurements and cardiopulmonary performance, are measured. Additionally, data regarding dietary intake and physical activity, as well as medical and family history, are collected through a questionnaire. Participants’ risk levels for several lifestyle conditions are then calculated, and, based on this risk stratification, participants follow a 16-week digital coaching program. The blended coaching combines human coaching and e-health to address the lifestyle domains (nutrition, activity, stress management, sleep, and other health behaviours) and is rooted in the Fogg Behavior Model [26]. The digital human coaching is 1-on-1 and a combination of chat- and call-based interactions. Through this approach, participants are provided peer-support and motivation, coached how to acquire and maintain healthy habits, learn how to overcome barriers encountered during behavior change, and receive tips/tricks on how to implement new behaviors into daily practice. In addition to the main coaching track on cardiometabolic risk management or mental health, participants with nutritional deficiencies at baseline received specific coaching on how to increase consumption of foods rich in the deficient micronutrients or to start supplementation where needed.

### 2.5. Statistical Analysis

Descriptive statistics were calculated to characterize the population at baseline, in terms of vitamin and mineral status, as well as food group consumption. Additional analyses were conducted in the group of patients who had an abnormal vitamin status at baseline and subsequently underwent a remeasurement. In this group, we calculated the changes in each of the relevant vitamins, minerals, and related markers. All categorical variables were reported as the number (percentage, %), and continuous variables as the mean and standard deviation (SD). For differences in categorical variables, the chi-square test was used, and analysis of variance tests were conducted for continuous variables. Statistical significance was set at *p* < 0.05 when not adjusted for multiple comparisons. The Pearson linear correlation factor, R, was used to assess the linear association between baseline food group consumption and micronutrient levels. Values of P for associations between food group consumption and mineral and vitamin levels were adjusted for multiple comparisons, with the P-value for significance set at 0.0038.

## 3. Results

The baseline nutritional intake data and mineral status were available for all 348 participants. We identified at least one nutritional imbalance in 301 individuals (86.5%). The baseline values and status for all markers are shown in Table 2.

### 3.1. Vitamin and Mineral Status

#### 3.1.1. B Vitamins

A total of 348 participants (153 men and 195 women) had a baseline measurement of vitamins B6, B12, and folate. Mean B6 levels were 74.5 nmol/L (SD 59.6), with 3 participants (0.9%) having B6 deficiency (<20 nmol/L), and 46 participants (13.2%) having low B6 (20–35 nmol/L). Of the remainder, 265 had a normal value (50–180 nmol/L), and 14 participants (4%) had excessively high levels (>180 nmol/L). For B12, 1 participant (0.3%) was deficient (<120 pmol/L), and 75 participants (21.6%) had low levels between 120 and 250 pmol/L (mean 198 pmol/L, SD 34). Lastly, 49 participants (14.1%) had folate deficiency (<10 nmol/L). There were no instances of B12 or folate deficiency anemia.

#### 3.1.2. Vitamin D

The same 348 participants underwent a baseline vitamin D measurement. Of these, 4 participants (1.2%) had severe deficiency (<25 nmol/L), and 79 participants (22.7%) were insufficient (25–50 nmol/L). In the remainder of the population, 178 participants (51.1%) were below normal (50–80 nmol/L), and 84 participants (24.1%) were in the desired range (80–150 nmol/L). Three participants (0.9%) were above the >150 nmol/L value, without a marked excess (mean 162 nmol/L, SD 9.5).

#### 3.1.3. Iron Status 

Iron deficiency (<10 µmol/L) was seen in 14 participants (4%), while iron excess was seen in 30 participants (8.6%). The remaining 287 participants were in the reference range (10–30 µmol/L), with a mean of 19.7 (5.2) µmol/L. Abnormal values in other markers of insufficient iron status were rare, with 5 (2.5%) men and 12 (6.2%) women having low ferritin (<40 µg/L and <15 µg/L, respectively), 5 of which also had low iron. High transferrin (>3.6%) was seen in six participants, one of which was also iron deficient. High transferrin saturation was found in 7 (2.2%) men and 47 (13.5%) women, the latter including 21 of the 30 with high iron. In total, six men (3.8%) and eight women (4.1%) had low hemoglobin (<8.5 and <7.5 mmol/L, respectively), with a mean cohort value of 9.4 (0.5) mmol/L for men, and 8.4 (0.6) mmol/L for women. Of these, three female participants had iron deficiency anemia.

### 3.2. Consumption of Major Food Groups and Supplements

**Figure 1:**
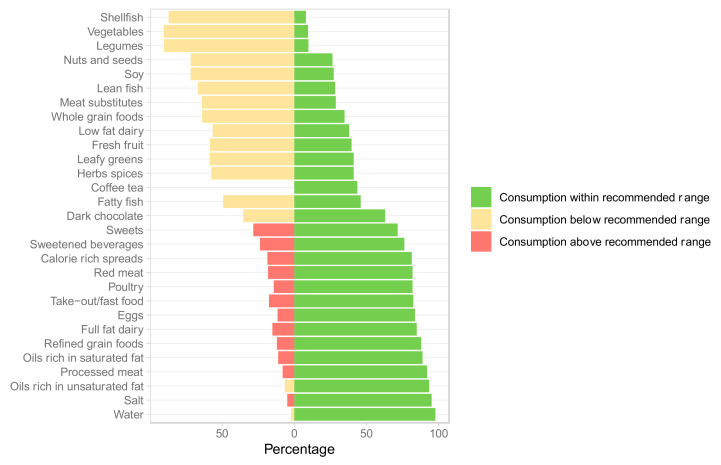
Consumption of different food groups at baseline (*n* = 348). Meat substitutes include all meat alternatives made from vegetarian or vegan ingredients, eaten as a replacement for meat.

Consumption of several healthy food groups was suboptimal (Figure 1). The largest gaps between reported insufficient and reported optimal intake were observed for vegetables (90.5% vs. 9.5%), leafy greens (59% vs. 41%), whole grains (65% vs. 35%), legumes (90.2% vs. 9.8%), fresh fruit (60% vs. 40%), and nuts and seeds (73.6% vs. 26.4%). In contrast, weekly intake of red and processed meat, refined grains, and sweetened beverages exceeded optimal levels in 81%, 91%, 88%, and 76% of the participants.

Use of any of the listed dietary supplements at baseline was reported by 136 (39%) participants, with 130 (96%) supplement users taking vitamin D, and 46 (34%) taking B vitamins. In supplement takers, vitamin D levels were higher (79 (25.7) vs. 61 (21.8) nmol/L), as were those of vitamin B6 (110 (86.3) vs. 69.1 (52.5)), folate (27.3 (11.2) vs. 17.7 (9.11)), and B12 (442 (166) vs. 60.5 (194.8)) (*p* < 0.001).

### 3.3. Impact of Food Group Consumption on Vitamin and Mineral Status

**Figure 2:**
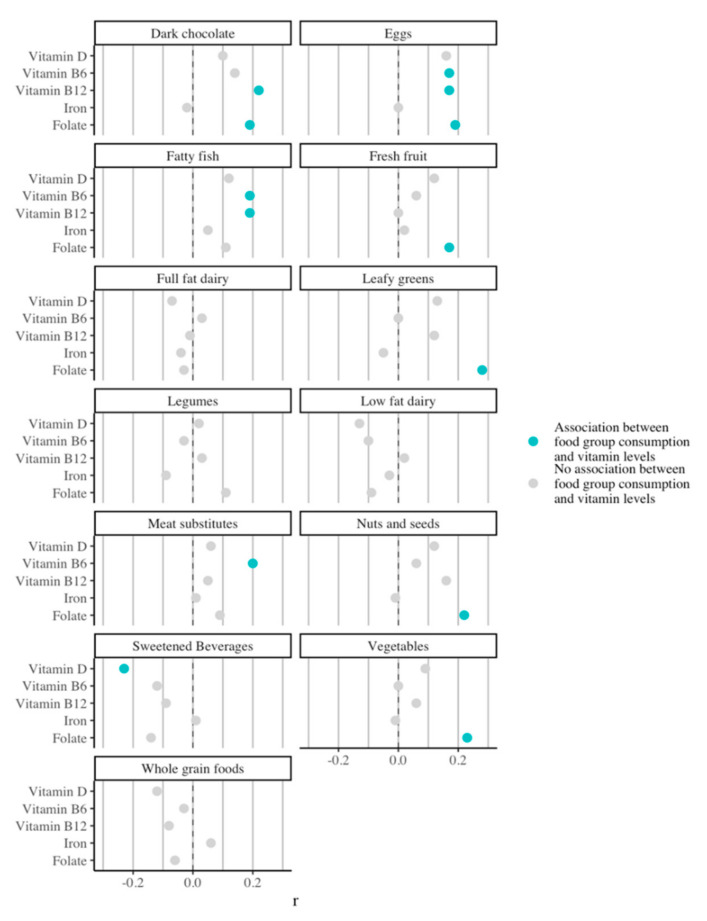
Association of consumption of different food groups with vitamin and iron status at baseline.

A high intake of eggs, meat substitutes, and different vegetables was positively associated with optimal micronutrient levels (Figure 2). Consuming more portions of meat substitutes and fatty fish was associated with higher vitamin B6 levels at baseline (R = 0.20 and 0.19, *p* < 0.002). Consuming more red meat, eggs, leafy greens, fresh fruit, vegetables, nuts and seeds, and dark chocolate was associated with higher folate levels (R = 0.28 to R = 0.17). Contrarily, consuming more processed meats was associated with lower folate levels (R = −0.20, *p* < 0.001).

### 3.4. Effect of a Digital Lifestyle Intervention on Nutrient Status and Food Group Consumption

#### 3.4.1. B Vitamins

Forty-nine participants had their vitamin B12 levels remeasured. Of the 20 with low B12 at baseline, 11 had improved at remeasurement, of which 9 achieved normal levels (mean 264 pmol/L, SD 78) (*p* = 0.006) (Table 3). For the remaining remeasured participants, B12 levels remained normal (*p* = 0.94). For vitamin B6, 10 participants were remeasured, of which 5 were at risk of deficiency at baseline, with a mean of 27.2 nmol/L (SD 2.1). None of the deficient subjects were remeasured. At remeasurement, four of the five at-risk individuals had returned to within normal levels, with mean levels increasing to 78.2 nmol/L (SD 39.4) (*p* < 0.05). In those with high B6 at baseline, five of the six participants showed drastic decreases and normalized their values, despite showing no changes in the mean value due to one individual whose levels further increased. Lastly, 38 participants remeasured folate. Of the 12 deficient at baseline, 9 showed improvement at remeasurement, and 7 achieved normal levels, with the mean values increasing from 8.3 nmol/L (0.9) to 12.7 nmol/L (6.8) (*p* = 0.05) (Table 3). None of the participants not taking supplements at baseline started taking supplements during the intervention. Box plots showing the changes in all participants are available in Appendix B.

#### 3.4.2. Vitamin D

Sixty-eight participants remeasured vitamin D. A total of 19 of the 22 participants with a deficiency at baseline improved, with vitamin D levels increasing at the group level from 39.7 nmol/L (SD 6.3) at baseline to 68 nmol/L (SD 23.5) at remeasurement (*p* < 0.001) (Table 3). The remaining 46 participants who were below the optimal level also showed an increase (+8.8 nmol/L, *p* = 0.0004), with 16 of them achieving normal levels. Of the 22 with a deficiency, 8 did not supplement at baseline but started supplementing, showing an increase from 34.6 nmol/L (SD 8.5) to 75.9 (SD 34.1) (*p* = 0.02). Those who did not supplement at baseline and did not start supplementation also showed an increase from 40.9 (SD 5.1) to 61.4 (SD 12.9) (*p* < 0.001).

#### 3.4.3. Iron Status 

Forty-five participants had their iron remeasured, of which one had a deficiency, three had iron overload, and eight had high iron and ferritin. At remeasurement, all had normalized their iron levels (mean 23 µmol/L, SD 7.7) (Table 3, *p* = 0.015). The three participants with iron deficiency anemia who would have received advice to visit their doctor for further treatment were not remeasured. With regard to other markers of iron status, the four participants at risk of iron deficiency anemia still with normal iron but low ferritin who were remeasured showed no changes. All four participants with high isolated ferritin returned to normal levels, with a decrease of 90 µg/L in the men, and 37 µg/L in the women (*p* = 0.04).

## 4. Discussion

In this study, we assessed the nutritional status of multiple vitamins and minerals in a Dutch general population cohort undergoing a preventive health screening and digitally enabled lifestyle intervention. We identified several nutritional imbalances to be more prevalent than expected and established some minor associations between normal nutrient levels and (in)sufficient consumption of certain food groups. In addition, we found a positive effect of a digital lifestyle intervention focused on nutritional habit coaching on the correction of the micronutrient imbalances.

Vitamin and mineral deficiencies affect people of all genders and ages across both lower- and higher-income countries, making them a problem of individual and societal relevance [15]. While the most prevalent MNDs do not directly cause specific diseases, they act as risk or exacerbating factors that impact the morbidity, mortality, and quality of life of those with infectious and chronic diseases [15]. With the increasing prevalence of chronic conditions in the general population, which in the Netherlands reached 50% in 2015, the link between subclinical deficiencies and NCCDs becomes all the more relevant [10,27]. The idea of taking preventive action on sub-normal vitamin levels, otherwise deemed fine in standard clinical care due to the absence of symptoms, has also become more widespread. For instance, the threshold for vitamin B6 deficiency varies across the literature, but recent international research and guidelines have lowered the threshold for action from 50 nmol/L to 30 nmol/L, as lower blood values are associated with a wide range of metabolic effects [28,29]. In our cohort, while vitamin B6 deficiency was rare, low B6 was found in more than 10% of the participants. After the intervention, mean blood values in this subpopulation were normalized through diet alone. Since no adverse events have been associated with high intakes of B6-rich foods, as opposed to the possible risks of excessive supplementation, this constitutes a good way to preemptively tackle lower B6 levels [30]. Data from Western countries show B12 and folate deficiencies to be more prevalent [31]. Indeed, we found that 24% of the participants were deficient in or had low B12, and 20% were folate deficient. The Dutch, and particularly Dutch women, have been found to have the lowest mean intake and mean intake from diet across Europe for both B vitamins, with as many as 46% of young women and women over 60 in the Netherlands consuming insufficient folate through diet [31]. Our analysis supports this, as the food groups known to be rich in B vitamins and which showed a positive association with optimal levels were precisely the food groups with the highest rate of insufficient intake. In our intervention, we showed that providing behavioral support to increase dietary intake can improve or correct low and deficient vitamin levels, with 55% of the participants with low B12, 80% with low B6, and 75% with low folate showing improvement. Similarly, while the Dutch population has been reported to have the highest daily intake of vitamin D in Western Europe across all age ranges owing to the widespread use of supplements, we encountered that 23% of individuals were deficient or insufficient, with a total of over 70% having suboptimal levels. This may be explained by findings from other previous analyses in a Dutch population, which indicated that the standard supplementation dose advised for maintaining vitamin D levels was insufficient to correct already existing deficiencies [32]. Despite a trend which is gaining traction in the Netherlands that suggests neither B12 nor vitamin D should be measured in primary care, as dietary consumption and sun exposure would be sufficient to prevent deficiency in “healthy subjects”, both these and our own data support that these measurements remain worthwhile. Both the limited sun hours in Northern European countries and their inhabitants‘ increasingly sedentary lifestyle do not promote vitamin D production from sun exposure, as previously reported in the literature and (anecdotally) also noted by participants themselves during the coaching intervention [33]. The key to restoring vitamin D levels is a combination of awareness, sufficient supplemental dosage, and the successful creation of the habit of using the supplement. With adequate behavioral support, it is not surprising that 86% of those with vitamin D deficiency at baseline showed improvement at remeasurement in this cohort.

With regard to iron status, we did not find significant abnormalities, which is in line with previous studies that showed iron intake in the Dutch population to be sufficient and abnormalities in iron status to be generally rare [33,34]. Interestingly, a small fraction of the participants were found to have high iron and ferritin with normal transferrin saturation. Unlike iron overload derived from hereditary hemochromatosis (which usually presents early in life and can lead to parenchymal damage in numerous organs), most non-genetic causes for abnormalities in iron status parameters often present asymptomatically and can therefore go undetected for long periods of time [35]. Two such entities are dysmetabolic hyperferritinemia (DH) and dysmetabolic iron overload syndrome (DIOS), which are closely associated with metabolic syndrome, hepatic steatosis, and insulin resistance [36,37,38]. We therefore further investigated whether these participants possessed any of the components of metabolic syndrome; this was not the case, leaving the reason for these abnormalities unexplained. Nonetheless, given the growing evidence for the co-occurrence of metabolic syndrome and DH or DIOS, including and combining cardiometabolic and iron status assessments in preventive lifestyle programs may be advised [37,39].

We believe this study also provides a contribution to two interesting debates that extend beyond its direct results. First, one debate is whether nutritional deficiencies should be addressed purely based on dietary changes or supplementation. On the one hand, it is arguably more cost effective, at an individual level, to meet nutritional requirements as part of daily food consumption [40,41]. For B vitamins and minerals, meeting the RDA through diet alone is sufficient to maintain normal blood levels and prevent deficiencies. Indeed, through the lifestyle intervention undergone after the baseline screening, all individuals with deficiencies other than vitamin D restored normal blood levels through changes to their diet. As such, we advocate for a diet-based approach as the standard approach to the correction of micronutrient imbalances in the general population. On the other hand, B vitamin deficiencies in vegetarian/vegan individuals are more prevalent as well as more difficult to correct without supplementation, and populations from Northern European countries show alarmingly high rates of vitamin D insufficiency without supplementation [42,43]. Therefore, supplementation remains a necessary alternative or complement for certain subpopulations. Interestingly, supplement use has been associated with positive lifestyle and nutritional health behaviors. While causality cannot be ascertained, this could indicate that individuals with good health behaviors choose supplementation as a low-effort, effective way of preventing or tackling specific nutrient deficiencies when already following good nutritional habits. Second, this study also contributes to the debate regarding the role of population screening for nutrient status, in a manner comparable to other population-wide programs such as for cardiovascular risk assessment and management. Evidence on the cost effectiveness of population screening for vitamin deficiencies is scarce, with some evidence from other Western countries failing to support population-wide nutritional status screening, for reasons of cost effectiveness [44,45,46]. However, our data support the idea that nutritional status screening has a place as part of personalized lifestyle programs, as it can provide a definitive assessment (rather than, for instance, estimates of micronutrient intake) of key micronutrients for chronic disease prevention, which these programs are ultimately geared at.

This study presents some limitations. First, given we targeted a broad nutritional assessment at the food group level, no validated questionnaire was available which could be used. While we provided clear, standardized descriptions of portion sizes to participants in the questionnaire assessment of dietary habits at the food group level, it is possible that the estimated food group consumption deviates to an extent from actual consumption. This is a known risk with all food frequency questionnaires, further validated or not. Second, while the reliability of our B12 measurements is high and overt B12 deficiency can be detected using direct measurement of serum B12, increased methylmalonic acid levels are a sensitive indicator of mild vitamin B12 deficiency, and elevated homocysteine levels may indicate vitamin B12 or folate deficiency without abnormal low levels of B12 [47]. By not having measured these two markers, we may have missed individuals with milder or incipient B12 deficiencies. Third, we did not quantify all supplement consumption beyond asking whether participants took individual supplements. Therefore, the associations between binary consumption of supplements and the associated micronutrient status are somewhat limited, and no conclusions regarding dose–response can be made. Finally, while we included an unselected sample of the general population, this cohort had a generally higher socioeconomic status and younger age distribution, which makes our findings not entirely generalizable to the entire Dutch population. Since remeasurement was voluntary, we also did not remeasure the entire cohort. Attrition rates in mHealth studies and real-world applications are known to be high, with previous research having identified that up to 80% of all participants in mHealth interventions may engage in only minimal use of these interventions, and that the lowest user dropout rate was 40%, even in a trial setting [48,49]. Together, these factors can introduce some bias in the analysis, since the subgroup which was remeasured is likely more motivated and therefore more likely to engage in and benefit from lifestyle interventions.

## 5. Conclusions

Micronutrient deficiencies of easily obtainable vitamins through diet or supplementation such as B vitamins and vitamin D were more prevalent than expected in a Western country—the Netherlands. For B vitamins, this can partly be explained by insufficient consumption of food groups rich in these vitamins such as leafy greens and other vegetables and legumes for folate, and enriched foods and healthy animal products for vitamin B12. An optimal micronutrient status is vital for the prevention of numerous chronic conditions, and the observed lower B and D vitamin levels in an otherwise healthy population highlight the potential value of general health screening programs coupled with coaching on nutrition and other lifestyle and health habits for the correction of micronutrient imbalances and advancement of preventive health. Our preliminary results in the segment of the cohort that underwent a remeasurement after participating in a subsequent digitally enabled lifestyle intervention show that many of these imbalances were corrected.

## Figures and Tables

**Table 1:** Vitamin, mineral, and iron status marker thresholds.

Marker (Unit)	Reference Range
Vitamin B6 (nmol/L)	Deficiency: <20 Low: 20–30 Normal: 30–180 High: >180
Folate (nmol/L)	Low: <10 Normal: ≥10
Vitamin B12 (pmol/L)	Deficiency: <120 Low: 120–250 Normal: ≥250
Vitamin D (nmol/L)	Deficiency: <25 Insufficiency: 50–80 Normal: 80–180 Elevated: >180
Iron (µmol/L)	Deficiency: <10 Normal: 10–30 High: ≥30
Hemoglobin (mmol/L)	Men: 8.5–11 Women: 7.5–10
Ferritin (µg/L)	Men: 30–400 Women: 15–150
Transferrin (g/L)	2.0–3.6
Transferrin saturation (%)	15–45

**Table 2:** Baseline characteristics and vitamin and mineral status.

Marker or Characteristic	Baseline Status, Total Cohort (*n* = 348)
**Demographics**	
Sex (% female)	56%
Age (years, SD)	44.6 (11.1)
**Vitamin B6**	
Mean (SD), in nmol/L	74.5 (59.6)
Deficient	3 (0.9%)
Risk of deficiency	26 (7.5%)
Normal	282 (81%)
Excess	16 (4.6%)
**Folate**	
Mean (SD), in nmol/L	18.9 (9.9)
Deficient	52 (14.9%)
Normal	296 (85.1%)
**Vitamin B12**	
Mean (SD), in pmol/L	371 (193)
Deficient	2 (0.6%)
Insufficient	80 (23%)
Normal	266 (76.4%)
**Vitamin D**	
Mean (SD), in nmol/L	68 (25)
Deficient	4 (1.1%)
Insufficient	79 (22.7%)
Suboptimal	178 (51.2%)
Normal	84 (24.1%)
Excessive	3 (0.9%)
**Iron status**	
Mean iron (SD), in µmol/L	20.4 (6.9)
Iron deficiency anemia	3 (0.9%)
Overt iron deficiency	14 (4%)
Normal iron + low ferritin	12 (3.4%)
Normal iron	304 (87.4%)
Iron overload High iron and ferritin	3 (0.9%) 30 (8.6%)
Isolated high ferritin	40 (11.5%)

SD = standard deviation. Participants were characterized as deficient or having normal levels according to the thresholds defined in Table 1.

**Table 3:** Pre- and post-intervention values for vitamins and minerals, including changes in status.

Marker	Before Lifestyle Intervention (*n*) ^a^	After Lifestyle Intervention (*n*) ^a^	*p*-Value ^b^
**Vitamin B6**	*n* = 29	*n* = 5	
Mean (SD), in mmol/L	27.2 (2.1)	78.2 (39.4)	0.04
Improved	-	4 (80%)	
Normalized	-	4 (80%)	
**Folate**	*n* = 52	*n* = 12	
Mean (SD), in mmol/L	8.3 (1.7)	12.7 (6.8)	0.05
Improved	-	8 (67%)	
Normalized	-	7 (58%)	
**Vitamin B12**	*n* = 82	*n* = 20	
Mean (SD), in mmol/L	205 (32)	264 (78)	0.006
Improved	-	14 (70%)	
Normalized	-	9 (45%)	
**Vitamin D**	*n* = 68	*n* = 22	
Mean (SD), in mmol/L	40 (6)	68 (24)	<0.001
Improved	-	19 (86.4%)	
Normalized	-	3 (13.6%)	
**Iron status ^c^**	*n* = 45	*n* = 12	
Mean (SD), in mmol/L	34.6	23.2	<0.001
Improved	-	10 (83.3%)	
Normalized	-	9 (75%)	

^a^ The number of participants who participated in the intervention and received nutritional coaching (middle column) and of those who were subsequently remeasured (right-most column) differs per marker, as shown. ^b^ Paired t-test for difference in means. ^c^ Comparison including only participants with elevated iron levels at baseline.

**Figure A1:**
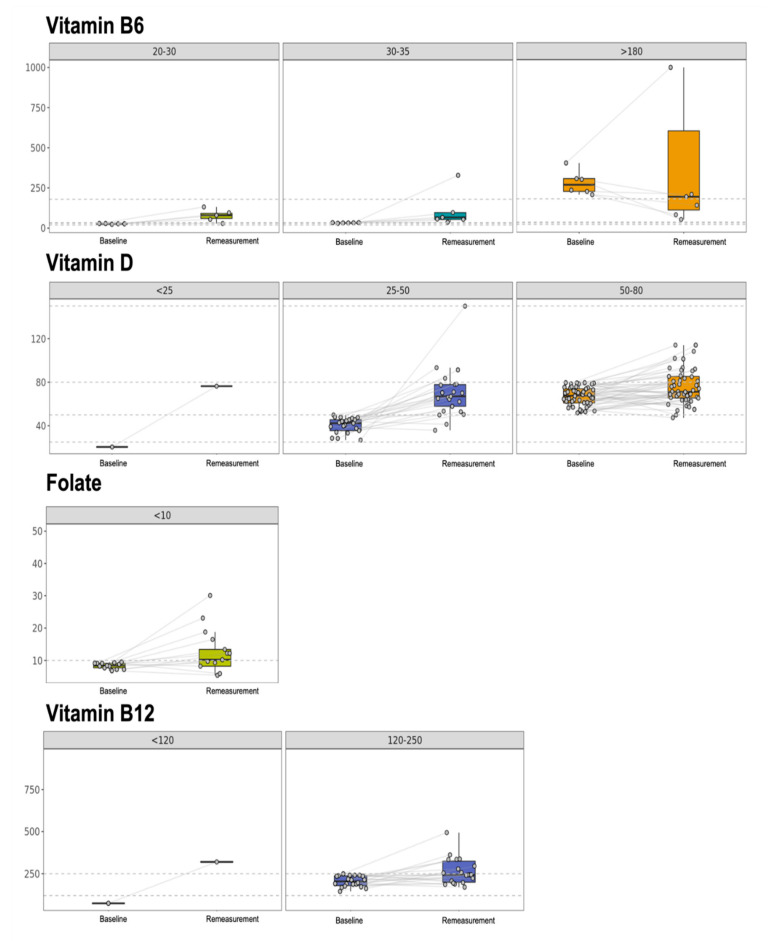
Box plots with pre- and post-intervention changes in B vitamins and vitamin D for the participants with abnormal baseline values.

## Data Availability

Data are available on request due to privacy restrictions. The data presented in this study are available on request from the corresponding author. As per the General Data Protection Regulation (GDPR), data are owned by the individuals undergoing the lifestyle programs, and Ancora Health B.V. receives consent to utilize these pseudoanonymized data for research.

## Appendix A

**Table A1:** Recommended thresholds for daily or weekly food group consumption, used as a guide for Figure 1. Values are based on Dutch guidelines for a balanced diet. Where unavailable, values based on expert opinion or from other sources were used.

Food Group	Lower Threshold (Portions)	Upper Threshold (Portions)
Shellfish	1 per week	3–6 per week
Vegetables	3 per day	No upper limit
Legumes	1 per day	No upper limit
Nuts and seeds	1 per day	2 per day
Soy *****	2 per week	3 per day
Lean fish	1 per week	6 per week
Meat substitutes *****	1 per day	6 per week
Whole grain foods	2 per day	4 per day
Low-fat dairy	1 per day	2 per day
Fresh fruit	2 per day	3 per day
Leafy greens	1 per day	No upper limit
Herbs and spices	1 per day	No upper limit
Coffee and tea	No lower limit	3 per day
Fatty fish	1 per week	6 per week
Dark chocolate	No lower limit	1 per day
Sweets	No lower limit	3–6 per week
Sweetened beverages	No lower limit	1–2 per week
Calorie-rich spreads	No lower limit	3–6 per week
Red meat	No lower limit	1–2 per week
Poultry	1 per week	3–6 per week
Take-out/fast food	No lower limit	<1 per week
Eggs	2 per week	1 per day
Full-fat dairy	No lower limit	3–6 per week
Refined grains	No lower limit	3–6 per week
Oils rich in saturated fat	No lower limit	1 per day
Processed meat	No lower limit	1–2 per week
Oils rich in unsaturated fat	No lower limit	3 per day
(Added) salt	No lower limit	1 per day
Water	6 glasses per day	No upper limit

***** Thresholds for these food groups are based on adjusted, non-standardized guidelines for vegetarian participants. Similarly, the thresholds for meats and dairy or fish did not apply if participants were vegetarian. For added salt, meals with added salt were counted.
